# Pain in midlife women: a growing problem in need of further research

**DOI:** 10.1186/s40695-022-00074-x

**Published:** 2022-05-05

**Authors:** Jelena M. Pavlović, Carol A. Derby

**Affiliations:** 1 Saul R. Korey Department of Neurology, Albert Einstein College of Medicine, Bronx, NY USA; 2 Montefiore Headache Center, Montefiore Medical Center, Bronx, NY USA; 3 Department of Epidemiology and Population Health, Albert Einstein College of Medicine, Bronx, NY USA

## Abstract

More than 10% of American adults experience some level of daily pain, and nearly 40 million (17.6%) experience episodes of severe pain annually. Women are particularly impacted by both episodic and chronic pain with higher prevalence and a greater level of pain-related disability compared to men. Midlife is a critical period for women during which the frequency of pain complaints begins to increase. Although pain is known to be influenced and controlled by sex hormones, it has not been widely recognized as a symptom of the menopausal transition outside of the menopause research community. The recent thematic series in this journal has specifically highlighted pain related conditions including rheumatoid arthritis, migraine and abdominal pain for which the significance among midlife women is not typically recognized. The studies presented in this thematic series present a small fraction of relevant, understudied questions regarding pain and its impact on women in midlife. Addressing the gaps in knowledge will require longitudinal studies that consider the emergence of pain symptomatology in relation to midlife trajectories of other symptoms and health determinants, as well as further study of new and emerging therapies.

Over the last two decades, the rising prevalence of pain and the related opioid epidemic have led to increased interest in better understanding the epidemiology and mechanisms of pain [1–3]. More than 10% of American adults experience some level of daily pain, and nearly 40 million (17.6%) experience severe levels of pain annually [2, 4, 5]. The prevalence of pain increases with age, in part due to the increasing prevalence of medical conditions which cause pain, such as arthritis and diabetes [6, 7]. Thus, the public health burden of pain is expected to increase dramatically with the demographic shift of the U.S. population to older ages. Furthermore, poorly controlled episodic pain can transform into chronic pain disorders (defined as 15 or more days of pain per month for 3 or more months), which are associated with significant disability and morbidity [2, 4, 8].

Women are particularly impacted by both episodic and chronic pain with higher prevalence and a greater level of pain-related disability compared to men [9, 10]. The pain related conditions with higher female preponderance affect a broad range of organs and body regions [11, 12]. These include fibromyalgia, chronic fatigue syndrome, complex regional pain syndrome, abdominal pain (irritable bowel syndrome), interstitial cystitis/bladder pain syndrome, chronic pelvic pain of uncertain origin, migraine and orofacial pain/TMJ disorders. These conditions tend to emerge during the reproductive years and often subside in late life although, despite their frequency, these conditions have been significantly understudied in all age groups.

Further, dysmenorrhea, a common pain phenomenon of reproductive age women has been suggested as a key contributing factor to the higher prevalence of pain disorders in women than men. Dysmenorrhea has been associated with enhanced pain sensitivity which may increase risk of developing pain conditions in midlife and beyond [13, 14].

Prior work has largely been limited to studies of sex differences in chronic pain while the primary biological mechanisms that mediate gender differences in pain expression have been understudied [9] and remain controversial [15]. Recently, the increasing trend in prevalence of pain and the current opioid epidemic have led to a new emphasis on better understanding mechanisms that underlie the disproportionate burden of pain disorders in women. Studies have explored sex hormones, immunologic markers, and functional brain imaging among others in order to elucidate possible factors related to sex differences [5, 8, 16, 17].

The existing literature suggests that midlife is a critical period for women during which the frequency of pain complaints begins to increase. Midlife women experience increasing prevalence of chronic pain symptoms (aches and pains, headache, genitourinary pain) even in the absence of newly diagnosed chronic conditions [18, 19]. Though the significant burden of pain symptoms in midlife women has long been recognized, few studies have addressed the epidemiology and mechanisms of pain in midlife women. Particularly lacking are longitudinal studies of pain trajectories across midlife. The critical need to fill this knowledge gap is underscored by the fact that the highest increase in opioid prescribing and opioid related overdose mortality has been among women in their 40s, 50s and 60s [3, 20].

The dearth of studies regarding pain in midlife women has contributed to under-diagnosis of specific pain disorders even in the setting of pain complaints. The consequence is poor management of pain in midlife women [21]. This is further compounded by the fact that midlife is also associated with increasing occurrence of a multitude of physical and psychological symptoms that can have complex interactions with pain complaints [19, 22]. Beyond the proximal effects of untreated pain, poor management of midlife pain may have long-term effects on later life health of women by limiting physical activity and functioning [23, 24]. Finally, pain presents a particular challenge for midlife women given its adverse impact on functioning during a time in the life-course that is marked by a high level of family and work demands [21].

For women, midlife is marked by the hormonal changes of the menopausal transition (M.T.) [25]. Though pain is known to be influenced and controlled by sex hormones, it has not been widely recognized as a symptom of the M.T. outside the menopause research community [18, 26]. Studies that have examined pain prevalence across the life span have not typically focused on the M.T [2, 3, 6]. Most work regarding symptoms associated with hormonal changes in the M.T. have focused primarily on the cardinal menopausal symptoms; vasomotor symptoms, or symptoms related to sleep and mood [22, 27, 28]. In comparison, relatively few studies have examined the occurrence of pain across the M.T. in relation to hormone changes. Further, studies which have examined pain during the M.T. have generally focused on pelvic pain or on non-specific, generic pain (e.g., broadly musculoskeletal or total pain), without distinctions between types of pain affecting different organs or parts of the body [18, 19, 26].

Current knowledge regarding the epidemiology of pain in midlife women is limited by several methodologic factors. Many studies are based on pain clinic populations which have well defined pain phenotypes but lack adequate consideration of important midlife characteristics including the M.T. Conversely, most studies of the M.T. include detailed hormonal and biomarker data but not specific characterization of pain symptoms. With the exception of the Study of Women’s Health Across the Nation (SWAN) and the Seattle Midlife Women’s Health Study, no longitudinal studies have examined changes in pain related complaints across the stages of the M.T. Another limitation in the field is related to challenges to adequately capturing pain complaints and operationalizing them as pain disorders within population based studies. As pain complaints are common in a high percent of the population, it is important to parse out not only the frequency but also the intensity of pain and interference of pain with activities of daily living [29]. Further, accurate and reliable characterization of pain complaints is complicated by temporal variations. Ascertainment of complaints multiple times per day, over multiple days may be required to optimize characterization of pain symptoms. Emerging methods that apply mobile technology such as smart phone apps are a promising strategy that should advance the study of pain mechanisms [29, 30].

Historically, the literature has focused on studies of pain defined broadly, or on sub-types of pain traditionally associated with the M.T. The recent thematic series in the journal Women’s Midlife Health (WMHL) specifically highlighted pain related conditions that while common and troublesome in midlife women are not typically recognized by the broader medical and research community as particularly burdensome at this time of life. Two of these articles are reviews that focus on rheumatoid arthritis [31] and migraine [32], pain causing conditions that can occur in either sex at any age. While not typically considered causes of midlife pain symptoms, both disorders tend to have a female predominance that peaks in midlife. These reviews indicate that although both conditions have been linked to hormonal changes in women there is not a clear understanding of the specific mechanisms that play a role in their pathophysiology and presentation. Symptom presentation of both rheumatoid arthritis and migraine appears to change with fluctuating hormonal states, and both generally improve in high and rising endogenous estrogen states (pregnancy) and worsen with hormonal fluctuations during the M.T [31, 32]. Nevertheless, treatment of both joint pain and migraine headache with exogenous estrogens has shown inconsistent results [33–35].

The under-recognition of rheumatoid arthritis (R.A.) in women during midlife and particularly the lesser recognized non-immunologic mechanisms and their complications which contribute either directly or indirectly to pain in R.A. are discussed in Chancay et al. [31]. Most R.A. treatment and management is focused on controlling the inflammatory components of the disease. The authors argue that recognizing the non-inflammatory contributors of R.A. pain, such as mechanical pain, fibromyalgia, and psychosocial factors creates an opportunity for more optimal treatment within the biopsychosocial model [31, 36]. The article highlights that midlife may be a critical period during which more complete “whole woman” approaches to managing R.A. may be important.

Another pain condition in need of a “whole woman” approach to diagnosis and treatment is migraine, a central nervous system condition in which the cardinal symptom, severe headache, typically begins in the teens and early 20’s. Although migraine is typically recognized as a leading pain condition in women during the reproductive years, the review by Pavlovic [32] emphasizes that migraine prevalence and symptom frequency peak during midlife. The hormonal regulation of migraine has been recognized since ancient times, although primarily with respect to the prominence of peri-menstrual attacks [37]. However, midlife and the M.T. in particular create a specific challenge for women with migraine as fluctuating hormonal cycles decrease the predictability of attacks. As noted in the review, this has important consequences for treatment and quality of life. Migraine treatment in midlife women is further complicated by the fact that treatment with exogenous estrogen containing compounds is controversial given that migraine has been associated with increased stroke risk [38, 39]. Consequently, strict guidelines limit use of estrogen containing contraceptives in women with migraine older than 35 years of age [40]. Given that cardiovascular risk rises in midlife and that many women are unable to tolerate or have unmet needs in migraine treatment, new emerging therapeutic approaches for migraine are particularly relevant for this age group.

The potential long-term pathophysiologic consequences of migraine were explored in the original study by Newman-Norlund et al. [41], illustrating the potential relevance of some emerging therapeutic modalities for active treatment of migraine in midlife women. Researchers assessed changes in cortical and subcortical brain volume on MRI in 12 patients with chronic migraine before and after treatment with sphenopalatine nerve blocks; a highly specialized in-office procedure. They observed potential correlations between changes in areas of cortex involved in pain processing and markers of pain pre- and post-treatment. Despite the small sample size, this is an important treatment study, which adds to our growing understanding of treatment-related brain changes associated with headache recovery in women with migraine. The fact that all women in the study had history of migraine for over a decade suggests the possibility of recovery even in long established disease states [41].

A fourth article in the thematic series addresses abdominal pain, a common pain symptom not typically perceived as a hallmark of midlife [42]. This study from the Seattle Midlife Women’s Health Study examines whether abdominal pain experienced at midlife is due to aging or to hormonal changes related to the M.T [42]. The authors assessed how changes in severity of abdominal pain change in relation to age, stage of the M.T., perceptions of stress and to both hormonal and stress related biomarkers. The Seattle study is uniquely poised to address this question, as it is one of the few established longitudinal cohorts of women that followed several hundred women for over two decades while they transitioned from the late reproductive phase of life into the postmenopausal years. The study has previously examined back and joint pain showing that they were predominantly related to age, but not to MT-related factors [43]. Consistent with the literature, the Callan et al. manuscript reported decreases in abdominal pain with increasing age [42]. They extended prior work showing that estrogen and testosterone are associated with lower severity of abdominal pain [42], while anxiety and perceived stress were associated with higher severity of abdominal pain.

Overall, the WMHL thematic series on pain illustrates several important themes regarding pain in midlife women. They show that pain is a common symptom among midlife women and that they experience pain in many domains beyond those commonly recognized as related to the M.T. While some sub-types of pain newly emerge in midlife, many common forms of pain, such as headache and abdominal pain, can worsen in midlife. The studies presented in this thematic series present a small fraction of relevant, understudied questions regarding pain in women’s midlife.

The midlife is clearly an essential time of not only hormonal transition but of emerging symptoms that likely impact future health outcomes. The influence of sex hormones on pain in midlife women is still under investigation. There is increasing recognition that psychological and social factors play an important role in pain. Further work is needed to examine both racial and ethnic disparities as well as socioeconomic factors related to the presentation of pain symptoms, and to diagnosis and management of pain disorders. Access to treatment in midlife women is of utmost importance given the pervasiveness and burden on a population level and the under-recognition in clinical practice [6, 8]. Furthermore, the treatment interruptions due to the ongoing COVID-19 pandemic are exacerbating these problems and creating a need for novel therapy alternatives using emergent technologies such as telemedicine. Incorporation of these new modalities into treatment plans of midlife women is of particular urgency [44, 45].

Addressing these gaps in knowledge will require longitudinal studies that consider the emergence of pain symptomatology in relation to midlife trajectories of other symptoms and health determinants, as well as further study of new and emerging therapies. In addition to development of traditional therapeutics, more holistic approaches are needed for effective study and treatment of pain in midlife women. Further, efforts are needed to better understand environmental and occupational risk factors for pain and interventions to ameliorate them. Finally, the association of pain with significant opioid related morbidity and mortality in midlife women [3, 20], suggests the need to better understand how pain symptomatology and its response to treatment change in midlife. Increased research regarding sex differences in response to treatment is essential. This will not only improve pain treatment for women but will further our understanding of the pathophysiology of pain. This increased knowledge may have ramifications for both sexes throughout the lifespan.

## Data Availability

Not applicable.
